# Multimodal image-guided surgery of HER2-positive breast cancer using [^111^In]In-DTPA-trastuzumab-IRDye800CW in an orthotopic breast tumor model

**DOI:** 10.1186/s13550-019-0564-z

**Published:** 2019-11-21

**Authors:** Marion M. Deken, Desirée L. Bos, Willemieke S. F. J. Tummers, Taryn L. March, Cornelis J. H. van de Velde, Mark Rijpkema, Alexander L. Vahrmeijer

**Affiliations:** 1 Department of Surgery, Leiden University Medical Center, Leiden, The Netherlands; 2 Department of Radiology and Nuclear Medicine, Radboud University Medical Center, Nijmegen, The Netherlands; 3 Department of Clinical Pharmacy and Toxicology, Leiden University Medical Center, Leiden, The Netherlands

**Keywords:** Multimodal imaging, HER2-positive breast cancer, [^111^In]In-DTPA-trastuzumab-IRDye800CW, Fluorescence, Image-guided surgery

## Abstract

**Background:**

Combining modalities using dual-labeled antibodies may allow preoperative and intraoperative tumor localization and could be used in image-guided surgery to improve complete tumor resection. Trastuzumab is a monoclonal antibody against the human epidermal growth factor-2 (HER2) receptor and dual-labeled trastuzumab with both a fluorophore (IRDye800CW) and a radioactive label (^111^In) can be used for multimodal imaging of HER2-positive breast cancer. The aim of this study was to demonstrate the feasibility of HER2-targeted multimodal imaging using [^111^In]In-DTPA-trastuzumab-IRDye800CW in an orthotopic breast cancer model.

**Methods:**

Trastuzumab was conjugated with *p*-isothiocyanatobenzyl (ITC)-diethylenetriaminepentaacetic acid (DTPA) and IRDye800CW-NHS ester and subsequently labeled with ^111^In. In a dose escalation study, the biodistribution of 10, 30, and 100 μg [^111^In]In-DTPA-trastuzumab-IRDye800CW was determined 48 h after injection in BALB/c nude mice with orthotopic high HER2-expressing tumors. Also, a biodistribution study was performed in a low HER2-expressing breast cancer model. In addition, multimodal image-guided surgery was performed in each group. Autoradiography, fluorescence microscopy, and immunohistochemically stained slices of the tumors were compared for co-localization of tumor tissue, HER2 expression, fluorescence, and radiosignal.

**Results:**

Based on the biodistribution data, a 30 μg dose of dual-labeled trastuzumab (tumor-to-blood ratio 13 ± 2) was chosen for all subsequent studies. [^111^In]In-DTPA-trastuzumab-IRDye800CW specifically accumulated in orthotopic HER2-positive BT474 tumors (101 ± 7 %IA/g), whereas uptake in orthotopic low HER2-expressing MCF7 tumor was significantly lower (1.2 ± 0.2 %IA/g, *p* = 0.007). BT474 tumors could clearly be visualized with both micro-SPECT/CT, fluorescence imaging and subsequently, image-guided resection was performed. Immunohistochemical analyses of BT474 tumors demonstrated correspondence in fluorescence, radiosignal, and high HER2 expression.

**Conclusions:**

Dual-labeled trastuzumab showed specific accumulation in orthotopic HER2-positive BT474 breast tumors with micro-SPECT/CT and fluorescence imaging and enabled image-guided tumor resection. In the clinical setting, [^111^In]In-DTPA-trastuzumab-IRDye800CW could be valuable for preoperative detection of (metastatic) tumors by SPECT/CT imaging, and intraoperative localization by using a gamma probe and fluorescence image-guided surgery to improve radical resection of tumor tissue in patients with HER2-positive tumors.

## Introduction

Breast cancer is the most common malignancy in women worldwide. Surgery is still the cornerstone of primary breast cancer treatment, and breast-conserving surgery followed by radiation therapy is increasingly applied with the same survival benefit as mastectomy [1]. Complete tumor resections are a challenge in breast-conserving therapy as local recurrence rates increase with positive resection margins [2, 3]. Patients with positive resection margins will have to undergo additional surgery, with an increased risk of complications, poorer cosmetic outcomes, and causing a delay in adjuvant treatment and higher healthcare costs.

Fifteen to 20% of breast cancer patients have a human epidermal growth factor-2 (HER2)-positive tumor - and HER2 is expressed at even higher levels (up to 50%) in patients with ductal carcinoma in situ [4, 5]. An increasing number of patients with HER2-positive breast cancer are receiving neoadjuvant therapy [6], making it difficult to distinguish between tumor and chemotherapy-induced fibrosis and to obtain clear resection margins [7, 8]. The intraoperative identification of malignant tissue after neoadjuvant therapy, of a non-palpable tumor or during a reoperation, is challenging. Currently, 16.4% of overall breast cancer patients in the Netherlands and up to 40% of patients worldwide have positive margin rates after breast-conserving surgery [9, 10]. In the USA, approximately one quarter of patients will undergo additional surgery after breast-conserving surgery [11]. HER2-enriched tumors even have the highest risk of local recurrence after breast-conserving surgery [12, 13]. Tumor-targeted molecular imaging could improve complete tumor resection by the visualization and detection of HER2-positive breast cancer in patients undergoing surgery.

Trastuzumab (Herceptin) is a humanized monoclonal antibody against the HER2 receptor and approved for (neo)adjuvant therapy and treatment of metastasized disease. The extracellular domain of HER2 on the plasma membrane is also a potential target for diagnostic biomarkers or imaging agents. In the clinic, trastuzumab is efficaciously labeled with gamma-emitter indium-111 (^111^In) for the identification of HER2-positive metastases by scintigraphy [14] and zirconium-89 (^89^Zr) for positron emission tomography (PET) [15, 16]. Current intraoperative detection of non-specific radiotracers by a gamma probe in breast cancer surgery is of value for tumor detection and (sentinel) lymph node mapping; however, these agents are not tumor-specific and not optimal for margin delineation [17].

Multimodal intraoperative imaging using targeted tracers in HER2-positive breast cancer patients may improve radical tumor resection, which is directly related to lower rates of recurrence [2, 3]. For the development of a dual-labeled tracer for image-guided surgery, we conjugated trastuzumab with both a radioactive (^111^In) and fluorescence (IRDye800CW) label and determined the biodistribution, performed micro-SPECT/CT and fluorescence imaging and image-guide surgery in an orthotopic HER2-positive breast cancer model.

## Methods

To determine the optimal tracer dose of [^111^In]In-DTPA-trastuzumab-IRDye800CW for multimodal imaging in vivo, a dose escalation biodistribution study was performed in mice with high HER2-expressing breast tumors. Multimodal image-guided surgery was performed in each group. Using the optimal dose, the biodistribution and imaging studies were subsequently performed in a low HER2-expressing breast cancer model. Finally, autoradiography and immunohistochemically stained slices of tumor tissue were compared for co-localization of tumor tissue, HER2 expression, fluorescence, and radiosignal.

### Orthotopic breast tumor model

All animal studies were approved by the national committee on animal experiments (CCD) and the institutional Animal Welfare Committee of the Radboud University Medical Center. BALB/c nu/nu female athymic mice (6–8 weeks old) were obtained from Janvier and were acclimatized to laboratory conditions for at least 1 week before injection of tumor cells. The mice were housed in ventilated cages with ad libitum access to sterilized food and water.

In this study, a HER2 high- and HER2 low-expressing orthotopic tumor model were used. BT474 and MCF7 cells (ATCC) were cultured in RPMI 1640 medium (Gibco) enriched with 10% fetal calf serum and 1% glutamine. BT474 or MCF7 cells were suspended in 30 μl of 50% complete RPMI 1640 medium with 50% Matrigel (BD Biosciences). Before inoculation, mice were brought under general anesthesia. A small incision was made unilaterally between the nipples of the inguinal group. The mammary gland was pulled out to expose the fat pad for injection of BT474 or MCF7 cells into the gland. Mice were orthotopically injected with 5 × 10^6^ BT474 cells or 500,000 MCF7 cells in 30 μl Matrigel/PBS. Additionally, an estradiol pellet (0.25 mg, Innovative Research of America) was subcutaneously implanted via the same inguinal incision. All animals developed breast tumors in 6 weeks. A total of 16 mice with the largest tumors and in the best physical condition were selected for the experiment, of which 14 mice with BT474 tumors were injected with 10 μg (*n* = 5), 30 μg (*n* = 5), or 100 μg (*n* = 4) of [^111^In]In-DTPA-trastuzumab-IRDye800CW; mice with MCF7 tumors (*n* = 2) were injected with 30 μg of [^111^In]In-DTPA-trastuzumab-IRDye800CW. Nuclear and optical imaging was performed once the tumors reached a volume of ~ 30 mm^3^.

### Synthesis DTPA-trastuzumab-IRDye800CW

The monoclonal antibody trastuzumab (Herceptin) was dissolved according to the manufacturers’ instruction and subsequently dialyzed with PBS for conjugation. Next, trastuzumab was conjugated with the fluorophore IRDye800CW-NHS ester (LI-COR Biosciences). The bifunctional chelator *p*-isothiocyanatobenzyl (ITC)-diethylenetriaminepentaacetic acid (DTPA) was purchased from Macrocyclics. 2.5 mg of trastuzumab was incubated in 0.1 M NaHCO_3_ (pH 8.5) at room temperature during 1 h with a 3-fold molar excess of the IRDye800CW-NHS ester. The pH of the phosphate buffer was adjusted to 9.5 to optimize the yield of the chelator conjugation reaction. Next, trastuzumab-IRDye800CW was incubated with a 10-fold molar excess of the ITC-DTPA in 0.1 M NaHCO_3_, pH 9.5 (room temperature, 1 h). The reaction mixture was transferred into a Slide-A-Lyzer cassette (molecular weight cut-off: 20,000 Da; Thermo Fisher Scientific) for the removal of unconjugated IRDye800CW and ITC-DTPA by extensive dialysis against 0.25 M NH_4_AcO, pH 5.5. The substitution ratio of the purified conjugate was determined on an Infinite Pro 200 (Tecan, Switzerland). The average substitution ratio of IRDye800CW molecules was 1.3 per antibody. For the optical properties of DTPA-trastuzumab-IRDye800CW and IRDye800CW, the absorption and emission spectra were measured using a microplate reader (Infinite Pro 200, Tecan). DTPA-trastuzumab-IRDye800CW was stored in the dark at 4 °C until radiolabeling.

### Radiolabeling of DTPA-trastuzumab-IRDye800CW

DTPA-trastuzumab-IRDye800CW was labeled with 2 MBq of ^111^In (Covidien, Petten, The Netherlands) per microgram of antibody in 0.5 M 2-(*N*-morpholino)ethanesulfonic acid (MES) buffer, pH 5.4 (2 times the volume of [^111^In]InCl_3_ solution) and incubated for 20 min at room temperature under metal free conditions. After incubation, unincorporated ^111^In was chelated by adding 50 mM ethylenediaminetetraacetic acid (EDTA) solution (to a final concentration of 5 mM).

Radiochemical purity was determined by instant thin-layer chromatography (ITLC) on silica gel strips (Agilent Technologies), using 0.15 M sodium citrate buffer, pH 6.0, as the mobile phase.

The immunoreactive fraction of dual-labeled trastuzumab was determined with BT474 cells as described by Lindmo et al. [18]. A serial dilution of BT474 cells was incubated with [^111^In]In-DTPA-trastuzumab-IRDye800CW for 1 h at room temperature. Subsequently, BT474 cells were centrifuged and analyzed in a gamma counter (2480 WIZARD^2^; PerkinElmer).

### Multimodal imaging and image-guided resection

Before intravenous injection, the injection volume of [^111^In]In-DTPA-trastuzumab-IRDye800CW was adjusted to 200 μl per mouse with 0.5% BSA/PBS. Groups of mice bearing a BT474 tumor were injected intravenously in the tail vein with 0.2 ml 0.5% BSA/PBS containing 10 μg (*n* = 5), 30 μg (*n* = 5), or 100 μg (*n* = 4) of [^111^In]In-DTPA-trastuzumab-IRDye800CW (11-13 MBq/mouse for SPECT/CT imaging and 0.7 MBq/mouse for biodistribution). The MCF7 tumor-bearing mice (*n* = 2) were injected with the optimal dose determined by the biodistribution of [^111^In]In-DTPA-trastuzumab-IRDye800CW in mice with BT474 tumors. For the feasibility of multimodal imaging, whole-body SPECT/CT and fluorescence images were obtained in 2 mice per group 2 days after injection. SPECT/CT images were acquired using the small animal U-SPECT-II/CT-system (MILabs, The Netherlands), using a 1.0-mm-diameter pinhole collimator tube. The total scanning time was 50 min per mouse for SPECT acquisitions, followed by 5-min CT acquisitions (spatial resolution, 160 μm; 65 kv; 612 μA) for anatomic reference. Scans were reconstructed with MILabs reconstruction software (version 34i).

After SPECT/CT imaging, mice were euthanized using O_2_/CO_2_ suffocation. Fluorescence imaging was performed before and after resection of tumors with the IVIS Lumina II closed-cabinet fluorescence scanner (Xenogen VivoVision; Caliper Life Sciences, USA). For all fluorescence imaging, the acquisition time was 1–5 min with the following parameters: F/stop 2, excitation filter 745 nm, background correction excitation 640 nm, emission filter indocyanine green (810–885 nm), autofluorescence 675 nm, field of view C, and binning medium. After removing the inguinal skin, imaging was repeated to expose the underlying tumor. Furthermore, all visible tumors were resected followed by fluorescence imaging to exclude residual disease.

### Biodistribution

The biodistribution of the radioactive label was determined for blood, muscle, heart, lung, spleen, pancreas, kidney, liver, stomach, duodenum, and tumor of all mice 48 h post-injection of [^111^In]In-DTPA-trastuzumab-IRDye800CW. The activity of ^111^In in the resected organs and tumors was measured in a gamma counter (2480 WIZARD^2^; PerkinElmer, USA). To correct for radioactive decay, samples were measured along with standards of the injected activity (in triplicate). The tissue accumulation of [^111^In]In-DTPA-trastuzumab-IRDye800CW was expressed as percentage injected activity per gram of tissue (%IA/g). Values are represented as mean uptake ± standard deviation (SD). Statistical analyses were performed using SPSS Statistics 23.0 (IBM). In the dose escalation study, a one-way ANOVA test with post hoc Bonferroni correction was performed to test for significant differences in tumor accumulation between the different dose levels. To compare the tumor-to-blood ratios and tumor uptake (%IA/g) in the high- and low-expressing tumor model, Student’s *t* test was performed. An *α*-value of 0.05 was used in all analyses; *p* < 0.05 was considered significant.

### Autoradiography and immunohistochemical analysis

Formalin-fixed, paraffin-embedded sections of all breast tumors cut in 5 μm were prepared for autoradiographic analysis (Aida Image Analyzer, version 4.21, Elysia-Raytest) after 2 weeks of exposure to a phosphor imaging plate and developed using the Typhoon FLA 7000 phoshor imager (GE Healthcare Life Sciences). Fluorescence imaging of the breast tumor sections was performed with an Odyssey Clx (LI-COR Biosciences) flatbed scanner (recording time 30 min, 800 nm channel, focus 1.0 mm). Subsequently, sections were stained for HER2 and standard H&E staining was performed.

## Results

**Fig. 1:**
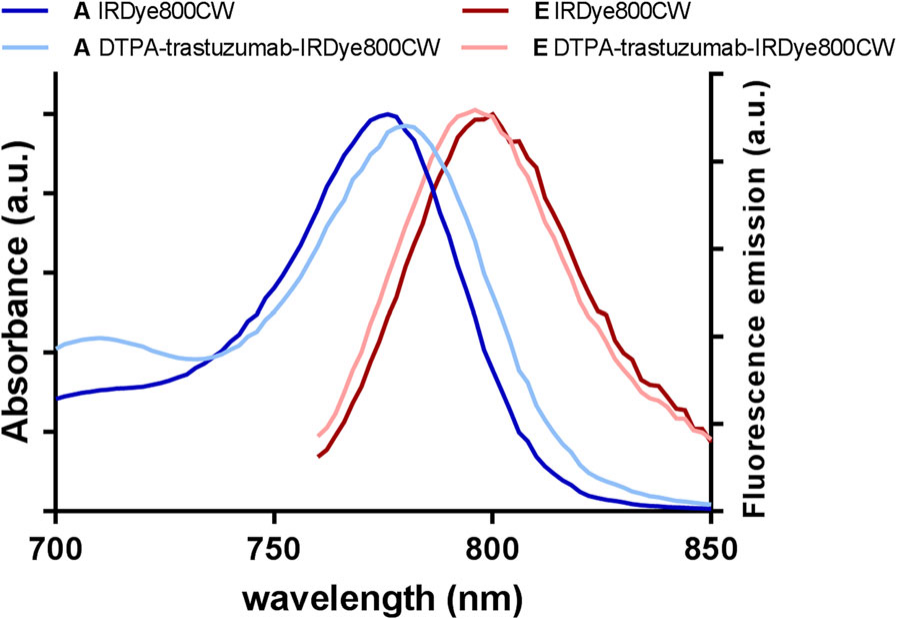
Absorption (A) and fluorescence emission (E) of IRDye800CW and DTPA-trastuzumab-IRDye800CW

[^111^In]In-DTPA-trastuzumab-IRDye800CW used in all studies had a high radiochemical purity (> 99%) and immunoreactivity (> 80%), and optical properties similar to the fluorophore IRDye800CW. The absorbance and fluorescence spectra of both IRDye800CW and DTPA-trastuzumab-IRDye800CW are shown in Fig. 1.

### Biodistribution

**Fig. 2:**
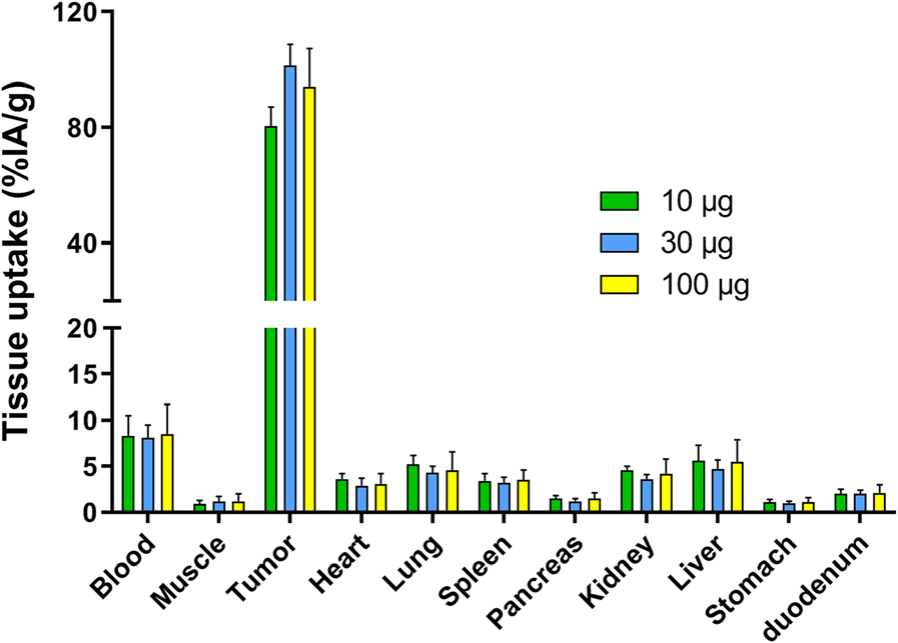
Biodistribution of 10 μg (*n* = 5, green), 30 μg (*n* = 5, blue), and 100 μg (*n* = 4, yellow) dual-labeled trastuzumab in mice with orthotopic BT474 tumors 48 h after administration. Tumor uptake after injection of 30 μg [^111^In]In-DTPA-trastuzumab-IRDye800CW was the highest %IA/g, while there was no significant difference to the 10 and 100 μg dose levels. Tissue uptake is expressed as %IA/g. Values represent mean ± SD

First, the optimal [^111^In]In-DTPA-trastuzumab-IRDye800CW dose 48 h after injection was determined in a dose escalation study in HER2 high-expressing tumors. Tumor-to-blood ratios of mice with BT474 tumors that received 10, 30, or 100 μg of [^111^In]In-DTPA-trastuzumab-IRDye800CW were 10 ± 3, 13 ± 2, and 11 ± 8, respectively. The average weight of the resected BT474 tumors in this orthotopic model was 0.03 g. Tumor uptake of [^111^In]In-DTPA-trastuzumab-IRDye800CW in BT474 tumors was 81 ± 7 %IA/g, 101 ± 7 %IA/g, and 94 ± 13 %IA/g after injection of 10, 30, and 100 μg dual-labeled trastuzumab, respectively. Tumor uptake after injection of 30 μg [^111^In]In-DTPA-trastuzumab-IRDye800CW was higher compared with tumor uptake after injection of 10 or 100 μg, but this was not significant. Accumulation of the different doses of dual-labeled trastuzumab was low in the liver (6 ± 2 %IA/g, 5 ± 1 %ID/g, and 6 ± 2 %IA/g for 10, 30, and 100 μg of [^111^In]In-DTPA-trastuzumab-IRDye800CW). The biodistribution results of 10, 30, and 100 μg [^111^In]In-DTPA-trastuzumab-IRDye800CW in BT474 tumors are summarized in Fig. 2.

**Fig. 3:**
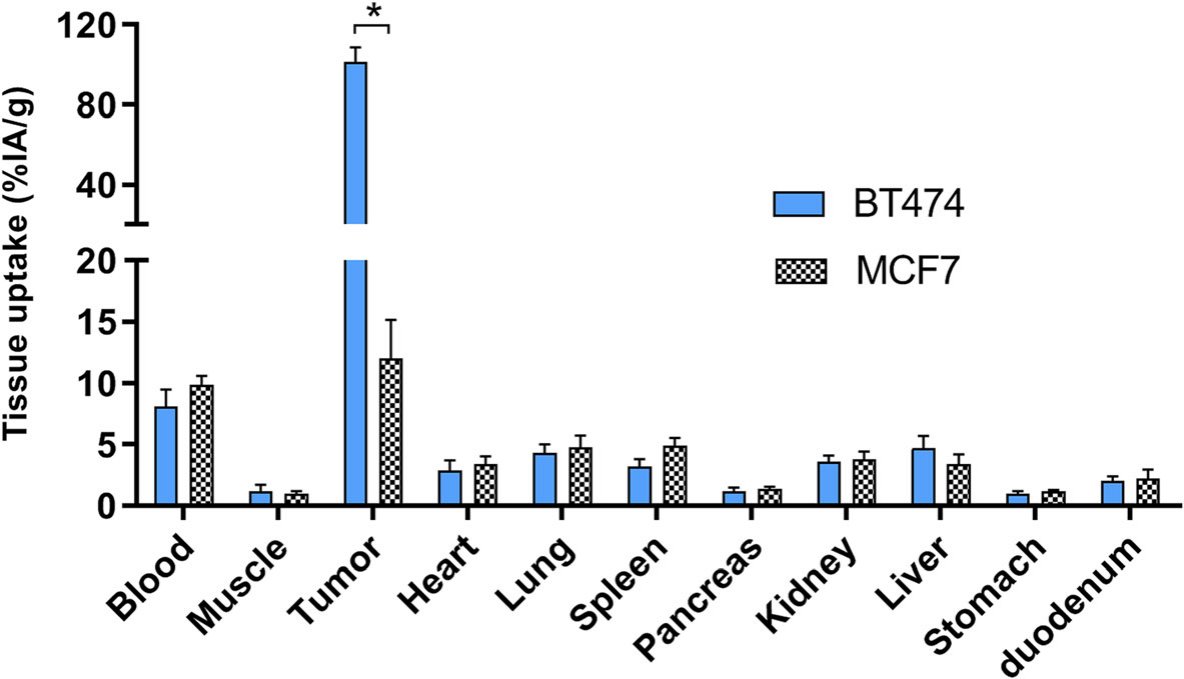
Biodistribution of 30 μg [^111^In]In-DTPA-trastuzumab-IRDye800CW 48 h after injection in orthotopic MCF7 tumor-bearing mice (black and white blocks), compared with uptake in BT474 tumors (blue). Tumor uptake in MCF7 tumors was significantly lower in comparison with uptake in BT474 tumors. Values represent mean ± SD. *Tumor uptake *p* < 0.0001

Based on these results, a 30 μg dose of [^111^In]In-DTPA-trastuzumab-IRDye800CW in BT474 tumors was chosen as the dose for the subsequent studies in MCF7 tumors (average weight of 0.03 g). The tumor-to-blood ratio of HER2 low-expressing MCF7 xenografts was 1.2 ± 0.2 at 48 h after injection of 30 μg [^111^In]In-DTPA-trastuzumab-IRDye800CW, which was significantly lower than in BT474 tumors (13 ± 2, p = 0.007). Tumor uptake in HER2 low-expressing tumors was significantly lower in comparison with uptake in HER2 high-expressing tumors (12 ± 3 %IA/g vs 101 ± 7 %IA/g, *p* < 0.0001). The biodistribution of 30 μg [^111^In]In-DTPA-trastuzumab-IRDye800CW in MCF7 tumors was compared with BT474 tumors in Fig. 3.

### Multimodal imaging and image-guided resection

**Fig. 4:**
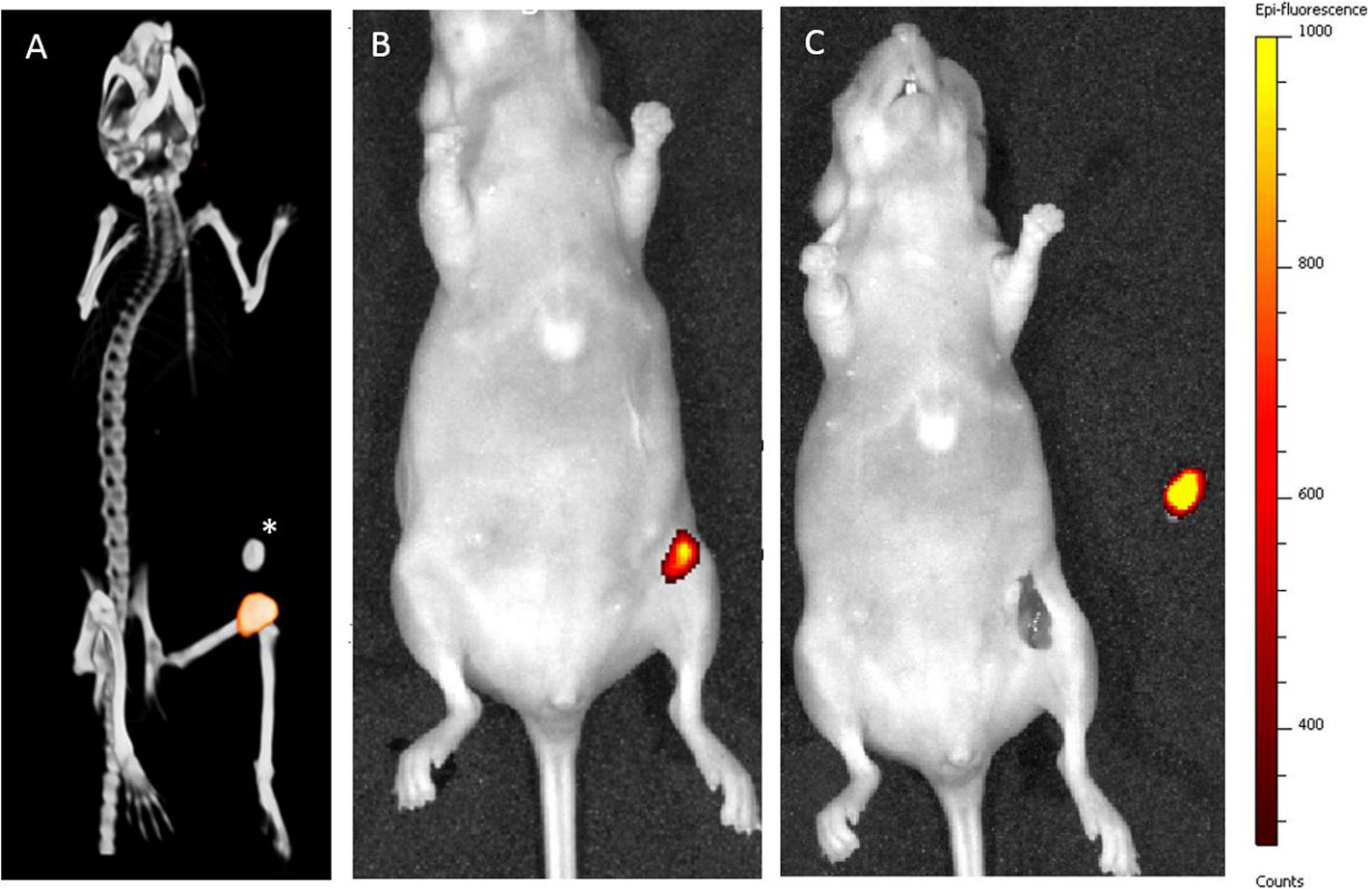
As an example a SPECT/CT image (supine position) of a mouse bearing a BT474 tumor (**a**), 48 h after injection of 30 μg [^111^In]In-DTPA-trastuzumab-IRDye800CW, showing high uptake in the tumor. Fluorescence images before and after resection of the tumor (**b**, **c**), with a selected epi-fluorescence scale of 300–1000 counts. *Implanted estradiol pellet

**Fig. 5:**
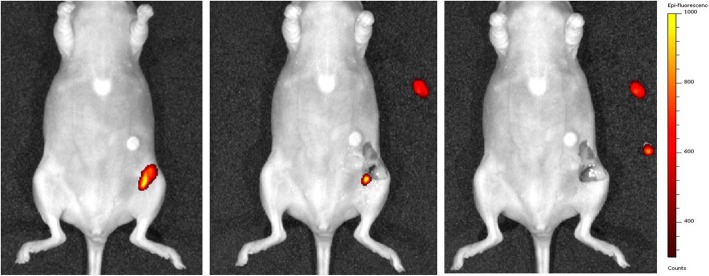
After injection of 10 μg [^111^In]In-DTPA-trastuzumab-IRDye800CW in a mouse bearing a BT474 tumor, fluorescence imaging identified an additional tumor lesion not identified during resection by white light. After resection of the second tumor, no further fluorescence signal was visible

Orthotopic breast tumors were clearly visualized by multimodal imaging 48 h after injection of [^111^In]In-DTPA-trastuzumab-IRDye800CW. Uptake of [^111^In]In-DTPA-trastuzumab-IRDye800CW was detected by micro-SPECT/CT imaging in BT474 breast tumors in all mice that were injected with either 10 μg, 30 μg, or 100 μg of the dual-labeled tracer (Fig. 4). Small-animal SPECT/CT imaging showed high uptake of dual-labeled trastuzumab in BT474 orthotopic breast tumors and no uptake in the liver or other organs. Following micro-SPECT/CT assessment, the BT474 tumors were first superficially identified by near-infrared fluorescence imaging. After resection of the inguinal skin, the tumors could be localized macroscopically and were detected with fluorescence imaging. Subsequently, the inguinal area was imaged after resection of the tumor to confirm radical surgical resection. Figure 4 shows an example of fluorescence imaging before and after resection of the tumors. However, in one mouse, a second smaller BT474 tumor, not identified during resection by white light, was revealed with fluorescence imaging (Fig. 5). After resection of this second tumor, no further fluorescence signal was visible. The presence of malignant cells in the second resected tumor was confirmed by histological assessment.

Accumulation of 10, 30, and 100 μg [^111^In]In-DTPA-trastuzumab-IRDye800CW could be visualized in BT474 tumors using the in vivo fluorescence imager with a selected epi-fluorescence scale of 300–1000 counts, whereas injection of 30 μg ^111^In-DTPA-trastuzumab-IRDye800CW in mice with MCF7 tumors showed no visible fluorescence signal on this selected scale (Additional file 1).

### Autoradiography and histological staining

**Fig. 6:**
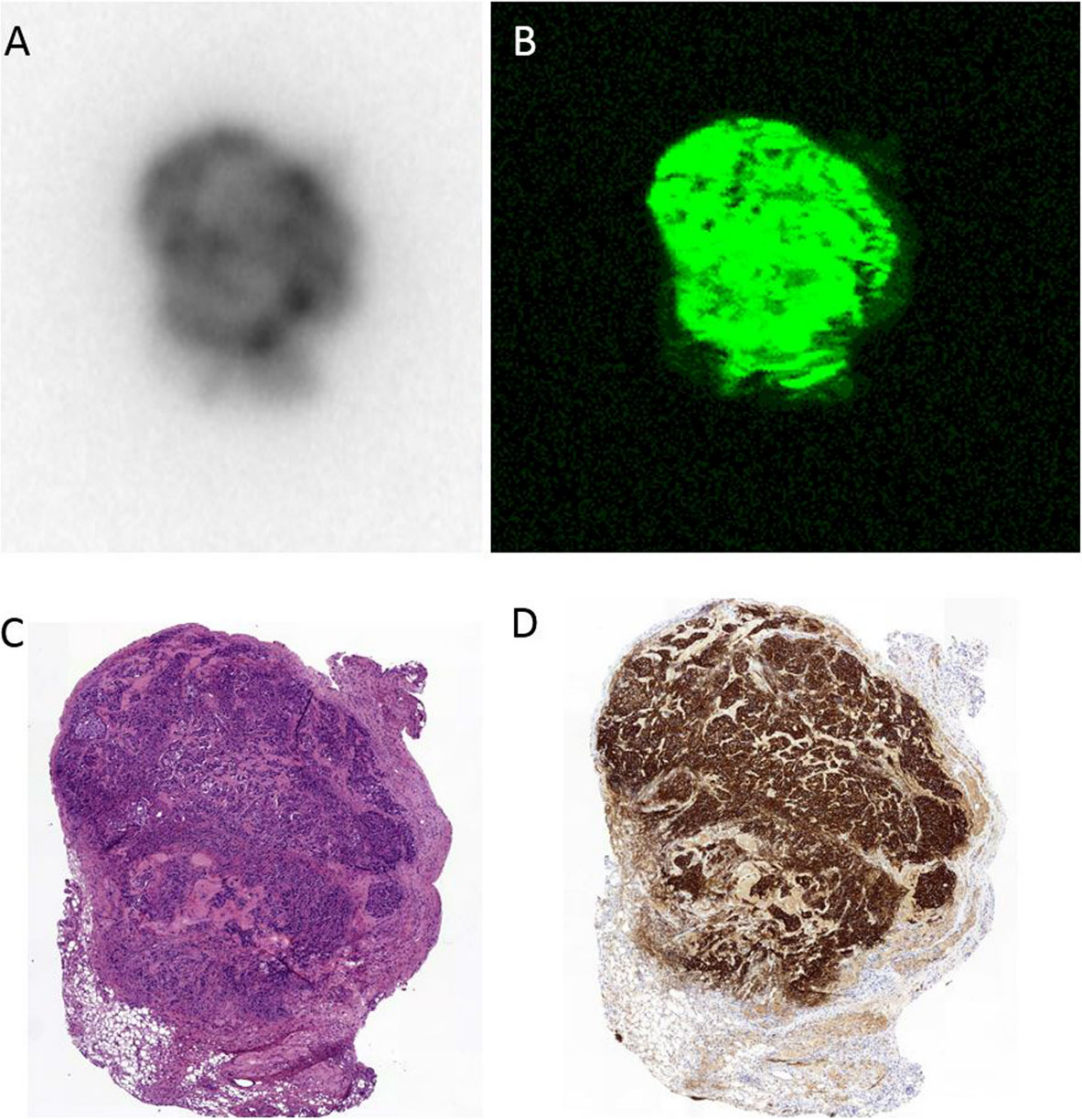
Autoradiography (**a**), fluorescence imaging (**b**), and immunohistochemistry (hematoxylin and eosin (**c**) and HER2 (**d**)) of 5-μm paraffin-embedded breast tumor sections confirmed fluorescence and radioactive signals co-localized with HER2-expressing tumors

Microscopic analysis of tumor tissue confirmed corresponding patterns of radioactive and fluorescence signal lesions with HER2 high-expressing tumors. A high level of HER2 expression was present in BT474 tumors as confirmed by immunohistochemistry and a low level of receptor expression was seen in MCF7 tumors. The distribution of the radioactive and fluorescence signals of [^111^In]In-DTPA-trastuzumab-IRDye800CW in tissue sections of BT474 tumors by autoradiography and flatbed fluorescence imaging showed co-localization with HER2 expression (Fig. 6).

## Discussion

This study shows feasibility of multimodal image-guided surgery using dual-labeled trastuzumab in an orthotopic HER2-overexpressing tumor model. The accumulation of [^111^In]In-DTPA-trastuzumab-IRDye800CW in HER2-overexpressing BT474 tumors was high and specific. Furthermore, the biodistribution of the dual-labeled tracer could be determined quantitatively with high accuracy and showed low uptake in other organs. High tumor-to-blood ratios were found at the three different dose levels. The uptake of [^111^In]In-DTPA-trastuzumab-IRDye800CW after 48 h (> 80 %IA/g) in high HER2-expressing tumors and the tumor-to-blood ratios were relatively high in comparison with tumor uptake of other dual-labeled tracers [19, 20], which could indicate high detectability of the dual-labeled trastuzumab in breast tumors.

Since the biodistribution of [^111^In]In-DTPA-trastuzumab was extensively determined previously in HER2-positive tumor-bearing mice, with the most pronounced uptake 2 days after injection [21], our biodistribution and imaging experiments were performed at one time point. Using the optimal dye to antibody ratio of 1 to 1.5 for labeling of antibodies with fluorophores [22], no negative effect on the in vivo behavior of [^111^In]In-DTPA-trastuzumab-IRDye800CW was expected. Our dual-labeled tracer even had considerably higher tumor uptake in comparison with the same concentration of a mono-labeled trastuzumab tracer in SKOV3 tumor cells (resp. > 80 %IA/g versus 16.3 %IA/g) [21]. Biodistribution studies in different subcutaneous animal models using dual-labeled trastuzumab have been described earlier, but with relatively low tumor accumulation and insufficient tumor-to-tissue ratios for further translation [23, 24]. However, a direct comparison of mono-labeled or other dual-labeled trastuzumab conjugates is difficult due to differences in exact conditions of conjugation, cell lines, and tumor models. The difference in chelator and dye-to-antibody ratio of trastuzumab in combination with our chosen random conjugation strategy may partially describe the different biodistributions. At a dye-to-antibody ratio of 2 or higher, the uptake of a dual-labeled antibody in the liver may increase, whereas the uptake in the tumor decreases [22]. Tumor uptake may also decrease with increasing chelator-to-antibody ratios. However, most chelating agents can be conjugated to antibodies in a ratio up to approximately 5:1 without having significant detrimental effect on biodistribution of the antibody [25–27]. To mimic the natural microenvironment of breast cancer, our biodistribution and imaging studies were not performed in a subcutaneous model but in an orthotopic breast cancer model. This could also contribute to the higher tumor uptake of our [^111^In]In-DTPA-trastuzumab-IRDye800CW in BT474 breast tumors. To date, none of these previous dual-labeled trastuzumab agents has been successfully translated for clinical use.

In this study, a high accumulation of dual-labeled trastuzumab in breast tumors was seen, while accumulation in the surrounding tissue, especially the liver, was low. The highest remaining activity in blood could be explained by the relative short window of 48 h between injection and measurement of radioactivity and is also seen in other in vivo experiments with labeled trastuzumab [28]. The dose in vivo was determined by the highest %IA/g by absence of a significant difference between the dose groups. However, the dose of [^111^In]In-DTPA-trastuzumab-IRDye800CW for optimal imaging in humans requires a dose escalation study in patients.

There are several limitations to this study. It should be noted that the small size of the tumors was suboptimal and in the control group, a limited amount of animals was studied. In the mouse model of this study, an orthotopic humanized tumor was employed and imaging was performed after injection of a humanized antibody. In humans, normal tissue may also express HER2, which might compromise the quality of imaging. However, normal tissue has a low complement of HER2 membrane protein, whereas HER2-positive breast cancers can have an increase up to 40–100-fold in HER2 protein resulting in 2 million receptors expressed at the tumor cell surface [29, 30]. This high tumor-to-normal tissue contrast will presumably ensure minimal or low detection of radioactive and fluorescence signals in benign tissue. Perik et al. [14] showed earlier that [^111^In]In-DTPA-trastuzumab enables identification of HER2-positive breast tumors in patients and in even 13/15 patients, new tumor lesions were discovered. Among these lesions, a liver metastasis was described, with a higher uptake of [^111^In]In-DTPA-trastuzumab in the liver metastasis than normal liver tissue. Therefore, in clinical situations, it is possible that our dual-labeled trastuzumab could similarly detect liver and other metastases by SPECT/CT imaging. The latter may also be important for other tumors in the abdomen. Moreover, our dual-labeled trastuzumab tracer could also be used for detection of other tumors overexpressing the HER2 receptor, such as gastric cancer, esophageal cancer, and bladder cancer [31].

After real-time intraoperative detection of breast cancer using non-specific fluorescence probes such as methylene blue [32] and indocyanine green [33], Lamberts et al. [34] introduced in 2017 the VEGF-A targeting antibody bevacizumab-IRDye800CW. Although ex vivo imaging of bevacizumab-IRDye800CW in tumors was feasible after bread-loaf slicing, in situ intraoperative tumor detection was still challenging. This highlights a limitation of near-infrared fluorescence imaging, which only has a penetration depth of up to a few millimeters in biological tissue as well as in adipose tissue around breast tumors, which implies that only superficial tumor lesions can be detected. Radio-guided surgery with gamma radiation has a high tissue penetration depth that allows accurate localization of tumors more deeply. Therefore, multimodal image-guided surgery, with the combination of a radiotracer for the detection of tumor tissue and optical tracers for accurate delineation and resection of tumor lesions, could improve surgical outcomes for breast cancer patients. The penetration depth (up to a few millimeter) of fluorophores as IRDye800CW is sufficient for the intraoperative identification of positive resection margins [35, 36], as Koller et al. demonstrated an 88% increase in the intraoperative detection rate of tumor-involved margins in primary breast cancer patients by fluorescence-guided surgery. In addition to an intravenous injection of a hybrid tracer for intraoperative imaging, whole-body SPECT/CT imaging could also be performed for non-invasive preoperative staging of the primary tumor, lymph nodes, and distant metastases. For example, preoperative SPECT/CT imaging of the primary breast tumor could contribute to the decision on surgery (breast-conserving treatment versus mastectomy). In patients, the visibility of tumor lesions detected on [^111^In]In-trastuzumab scintigraphy continued to increase up to the latest time point (168 h) after injection [37], indicating sufficient radioactive signals for intraoperative detection up to 7 days after injection of [^111^In]In-DTPA-trastuzumab-IRDye800CW, creating a time window for the planning of preoperative SPECT/CT imaging and intraoperative imaging. The principle of tumor-specific multimodal image-guided surgery has already been translated successfully into the clinic. Hekman et al. [38] demonstrated the feasibility of multimodal imaging by preoperative SPECT/CT, intraoperative gamma probe detection, and fluorescence imaging with [^111^In]In-DOTA-girentuximab-IRDye800CW in clear cell renal cell carcinoma patients.

In HER2-positive breast cancer patients, intraoperative tumor localization and delineation could be difficult, for example in patients with a residual tumor after neoadjuvant therapy, scheduled for a reoperation or resection of a non-palpable tumor. For these patients, multimodal image-guided surgery with [^111^In]In-DTPA-trastuzumab-IRDye800CW could of value. Post-neoadjuvant chemotherapy mastectomy specimens revealed fibrosis, necrosis, and inflammation, without grossly visible residual tumor beds [39, 40]. As a result of these changes in breast tissue, it can be difficult to intraoperatively identify residual disease and to perform an adequate radical breast-conserving resection after neoadjuvant chemotherapy. Therefore, preoperative image-guided localization techniques for non-palpable or residual breast lesions such as localization by guide-wire placement or implantation of iodine-labeled titanium seeds (I-125 seeds) are standard of care. Nevertheless, these current localization techniques are preoperatively invasive and only localize the tumor; accurate delineation of tumor tissue during a resection is not possible.

Patients with HER2-positive primary or metastatic breast cancer are increasingly treated with anti-HER2 therapy, including trastuzumab, in the neoadjuvant or adjuvant setting. Even though trastuzumab treatment decreased tumor uptake by around 20%, HER2 imaging with [^111^In]In-DTPA-trastuzumab was still feasible during or after trastuzumab treatment [37]. As a result of neoadjuvant treatment, loss of HER2-positive status in the residual tumor can occur, although around 90% of the patients still have a HER2 status of 2+ or 3+ with immunohistochemical analysis [41]. Therefore, [^111^In]In-DTPA-trastuzumab-IRDye800CW probably allows multimodal imaging of HER2-positive breast cancer in patients receiving neoadjuvant trastuzumab treatment, but further research is necessary.

## Conclusion

In conclusion, this study demonstrates the feasibility of multimodal image-guided surgery with [^111^In]In-DTPA-trastuzumab-IRDye800CW targeting HER2-expressing breast cancer in an orthotopic mouse model. In the clinical setting, [^111^In]In-DTPA-trastuzumab-IRDye800CW may allow preoperative detection of tumors by SPECT/CT imaging, subsequent intraoperative localization by gamma probe, and fluorescence image-guided surgery to improve radical resection of tumor tissue in patients with HER2-positive tumors.

## Supplementary information

**Additional file 1:** Orthotopic breast tumor model. During the period of tumor inoculation 8 mice developed abdominal skin lesions and had enlarged abdomens. After dissection of two of these mice, no clear indications were seen for these symptoms. The quantification of tumor uptake was unreliable in 2 mice because the tumor weight could not be determined accurately (below 0.01 gram). Two tumor measurements were left out of the analysis due to a substantial deviation of the average %IA/g: 6 %IA/g tumor uptake after injection of 10 μg and 30 %IA/g tumor uptake after injection of 30 μg. Both measurements were > 9 SD from the average in their group. One error could be explained by a substantially lower tumor weight and almost no HER2 expression (confirmed by immunohistochemistry); for both mice there was no difference in uptake of the tracer in other organs. **Figure S1.** Fluorescence images before and after resection of an orthotopic MCF7 tumor (A + B), 48 hours after administration of 30 μg [^111^In]In-DTPA-trastuzumab-IRDye800CW. The MCF7 tumor shows no fluorescence signal on the selected epi-fluorescence scale of 300-1000 counts.

## Data Availability

All data generated or analyzed during this study are included in this published article and its supplementary files.

## Abbreviations

%IA/g: Percentage injected activity per gram of tissue
ANOVA: Analysis of variance
CCD: National committee on animal experiments
CT: Computed tomography
DTPA: Diethylenetriaminepentaacetic acid
EDTA: Ethylenediaminetetraacetic acid
H&E: Hematoxylin and eosin
HER2: Human epidermal growth factor-2
PET: Positron emission tomography
SD: Standard deviation
SPECT/CT: Single photon emission computed tomography/computed tomography
VEGF: Vascular endothelial growth factor

## References

[1] Fisher B, Anderson S, Bryant J, Margolese RG, Deutsch M, Fisher ER (2002). Twenty-year follow-up of a randomized trial comparing total mastectomy, lumpectomy, and lumpectomy plus irradiation for the treatment of invasive breast cancer. N Engl J Med..

[2] Horst KC, Smitt MC, Goffinet DR, Carlson RW (2005). Predictors of local recurrence after breast-conservation therapy. Clin Breast Cancer..

[3] Mechera R, Viehl CT, Oertli D (2009). Factors predicting in-breast tumor recurrence after breast-conserving surgery. Breast Cancer Res Treat..

[4] Allred DC, Clark GM, Molina R, Tandon AK, Schnitt SJ, Gilchrist KW (1992). Overexpression of HER-2/neu and its relationship with other prognostic factors change during the progression of in situ to invasive breast cancer. Hum Pathol..

[5] Horimoto Y, Tokuda E, Arakawa A, Kosaka T, Saito M, Kasumi F (2011). Significance of HER2 protein examination in ductal carcinoma in situ. J Surg Res..

[6] Buzdar AU, Suman VJ, Meric-Bernstam F, Leitch AM, Ellis MJ, Boughey JC (2013). Fluorouracil, epirubicin, and cyclophosphamide (FEC-75) followed by paclitaxel plus trastuzumab versus paclitaxel plus trastuzumab followed by FEC-75 plus trastuzumab as neoadjuvant treatment for patients with HER2-positive breast cancer (Z1041): a randomised, controlled, phase 3 trial. Lancet Oncol..

[7] Bossuyt V, Provenzano E, Symmans WF, Boughey JC, Coles C, Curigliano G (2015). Recommendations for standardized pathological characterization of residual disease for neoadjuvant clinical trials of breast cancer by the BIG-NABCG collaboration. Ann Oncol..

[8] Cain H, Macpherson IR, Beresford M, Pinder SE, Pong J, Dixon JM (2017). Neoadjuvant therapy in early breast cancer: treatment considerations and common debates in practice. Clin Oncol (R Coll Radiol)..

[9] Pleijhuis RG, Graafland M, de Vries J, Bart J, de Jong JS, van Dam GM (2009). Obtaining adequate surgical margins in breast-conserving therapy for patients with early-stage breast cancer: current modalities and future directions. Ann Surg Oncol..

[10] Haloua MH, Volders JH, Krekel NM, Barbe E, Sietses C, Jozwiak K (2016). A nationwide pathology study on surgical margins and excision volumes after breast-conserving surgery: there is still much to be gained. Breast..

[11] Wilke LG, Czechura T, Wang C, Lapin B, Liederbach E, Winchester DP (2014). Repeat surgery after breast conservation for the treatment of stage 0 to II breast carcinoma: a report from the National Cancer Data Base, 2004-2010. JAMA Surg..

[12] Voduc KD, Cheang MC, Tyldesley S, Gelmon K, Kennecke H, Nielsen TO (2010). Breast cancer subtypes and the risk of local and regional relapse. J Clin Oncol..

[13] Jia H, Jia W, Yang Y, Li S, Feng H, Liu J (2014). HER-2 positive breast cancer is associated with an increased risk of positive cavity margins after initial lumpectomy. World J Surg Oncol..

[14] Perik PJ, Lub-De Hooge MN, Gietema JA, van der Graaf WT, de Korte MA, Jonkman S (2006). Indium-111-labeled trastuzumab scintigraphy in patients with human epidermal growth factor receptor 2-positive metastatic breast cancer. J Clin Oncol..

[15] Laforest R, Lapi SE, Oyama R, Bose R, Tabchy A, Marquez-Nostra BV (2016). [(89)Zr]Trastuzumab: evaluation of radiation dosimetry, safety, and optimal imaging parameters in women with HER2-positive breast cancer. Mol Imaging Biol..

[16] Dijkers EC, Oude Munnink TH, Kosterink JG, Brouwers AH, Jager PL, de Jong JR (2010). Biodistribution of 89Zr-trastuzumab and PET imaging of HER2-positive lesions in patients with metastatic breast cancer. Clin Pharmacol Ther..

[17] Holloway CM, Scollard DA, Caldwell CB, Ehrlich L, Kahn HJ, Reilly RM (2013). Phase I trial of intraoperative detection of tumor margins in patients with HER2-positive carcinoma of the breast following administration of 111In-DTPA-trastuzumab Fab fragments. Nucl Med Biol..

[18] Lindmo T, Boven E, Cuttitta F, Fedorko J, Bunn PA (1984). Determination of the immunoreactive fraction of radiolabeled monoclonal antibodies by linear extrapolation to binding at infinite antigen excess. J Immunol Methods..

[19] Lutje S, Rijpkema M, Franssen GM, Fracasso G, Helfrich W, Eek A (2014). Dual-modality image-guided surgery of prostate cancer with a radiolabeled fluorescent anti-PSMA monoclonal antibody. J Nucl Med..

[20] Rijpkema M, Oyen WJ, Bos D, Franssen GM, Goldenberg DM, Boerman OC (2014). SPECT- and fluorescence image-guided surgery using a dual-labeled carcinoembryonic antigen-targeting antibody. J Nucl Med..

[21] Lub-de Hooge MN, Kosterink JG, Perik PJ, Nijnuis H, Tran L, Bart J (2004). Preclinical characterisation of 111In-DTPA-trastuzumab. Br J Pharmacol..

[22] Rijpkema M, Bos DL, Cornelissen AS, Franssen GM, Goldenberg DM, Oyen WJ (2015). Optimization of dual-labeled antibodies for targeted intraoperative imaging of tumors. Mol Imaging..

[23] Wang X, Aldrich MB, Marshall MV, Sevick-Muraca EM (2015). Preclinical characterization and validation of a dual-labeled trastuzumab-based imaging agent for diagnosing breast cancer. Chin J Cancer Res..

[24] Sampath L, Kwon S, Ke S, Wang W, Schiff R, Mawad ME (2007). Dual-labeled trastuzumab-based imaging agent for the detection of human epidermal growth factor receptor 2 overexpression in breast cancer. J Nucl Med..

[25] Al-Ejeh F, Darby JM, Thierry B, Brown MP (2009). A simplified suite of methods to evaluate chelator conjugation of antibodies: effects on hydrodynamic radius and biodistribution. Nucl Med Biol..

[26] Shin IS, Lee SM, Kim HS, Yao Z, Regino C, Sato N (2011). Effect of chelator conjugation level and injection dose on tumor and organ uptake of 111In-labeled MORAb-009, an anti-mesothelin antibody. Nucl Med Biol..

[27] Kukis DL, DeNardo GL, DeNardo SJ, Mirick GR, Miers LA, Greiner DP (1995). Effect of the extent of chelate substitution on the immunoreactivity and biodistribution of 2IT-BAT-Lym-1 immunoconjugates. Cancer Res..

[28] Dijkers EC, Kosterink JG, Rademaker AP, Perk LR, van Dongen GA, Bart J (2009). Development and characterization of clinical-grade 89Zr-trastuzumab for HER2/neu immunoPET imaging. J Nucl Med..

[29] Kallioniemi OP, Kallioniemi A, Kurisu W, Thor A, Chen LC, Smith HS (1992). ERBB2 amplification in breast cancer analyzed by fluorescence in situ hybridization. Proc Natl Acad Sci U S A..

[30] Gutierrez C, Schiff R (2011). HER2: biology, detection, and clinical implications. Arch Pathol Lab Med..

[31] Yan M, Schwaederle M, Arguello D, Millis SZ, Gatalica Z, Kurzrock R (2015). HER2 expression status in diverse cancers: review of results from 37,992 patients. Cancer Metastasis Rev..

[32] Tummers QR, Verbeek FP, Schaafsma BE, Boonstra MC, van der Vorst JR, Liefers GJ (2014). Real-time intraoperative detection of breast cancer using near-infrared fluorescence imaging and Methylene Blue. Eur J Surg Oncol..

[33] Keating J, Tchou J, Okusanya O, Fisher C, Batiste R, Jiang J (2016). Identification of breast cancer margins using intraoperative near-infrared imaging. J Surg Oncol..

[34] Lamberts LE, Koch M, de Jong JS, Adams ALL, Glatz J, Kranendonk MEG (2017). Tumor-specific uptake of fluorescent bevacizumab-IRDye800CW microdosing in patients with primary breast cancer: a phase I feasibility study. Clin Cancer Res..

[35] Moore LS, Rosenthal EL, Chung TK, de Boer E, Patel N, Prince AC (2017). Characterizing the utility and limitations of repurposing an open-field optical imaging device for fluorescence-guided surgery in head and neck cancer patients. J Nucl Med..

[36] Koller M, Qiu SQ, Linssen MD, Jansen L, Kelder W, de Vries J (2018). Implementation and benchmarking of a novel analytical framework to clinically evaluate tumor-specific fluorescent tracers. Nat Commun..

[37] Gaykema Sietske B.M., de Jong Johan R., Perik Patrick J., Brouwers Adrienne H., Schröder Carolien P., Munnink Thijs H. Oude, Bongaerts Alphons H.H., de Vries Elisabeth G.E., Hooge Marjolijn N. Lub-de (2014). 111 In-Trastuzumab Scintigraphy in HER2-Positive Metastatic Breast Cancer Patients Remains Feasible during Trastuzumab Treatment. Molecular Imaging.

[38] Hekman MC, Rijpkema M, Muselaers CH, Oosterwijk E (2018). Hulsbergen-Van de Kaa CA, Boerman OC, et al. Tumor-targeted dual-modality imaging to improve intraoperative visualization of clear cell renal cell carcinoma: a first in man study. Theranostics..

[39] Park CK, Jung WH, Koo JS (2016). Pathologic evaluation of breast cancer after neoadjuvant therapy. J Pathol Transl Med..

[40] Sahoo S, Lester SC (2009). Pathology of breast carcinomas after neoadjuvant chemotherapy: an overview with recommendations on specimen processing and reporting. Arch Pathol Lab Med..

[41] Niikura N, Tomotaki A, Miyata H, Iwamoto T, Kawai M, Anan K (2016). Changes in tumor expression of HER2 and hormone receptors status after neoadjuvant chemotherapy in 21,755 patients from the Japanese breast cancer registry. Ann Oncol..

