# Supplementary figures and images for: Multimodal image-guided surgery of HER2-positive breast cancer using [111In]In-DTPA-trastuzumab-IRDye800CW in an orthotopic breast tumor model
Source: EJNMMI Res. 2019 Nov 21;9:98. doi: 10.1186/s13550-019-0564-z (PMC6872692; doi:10.1186/s13550-019-0564-z)

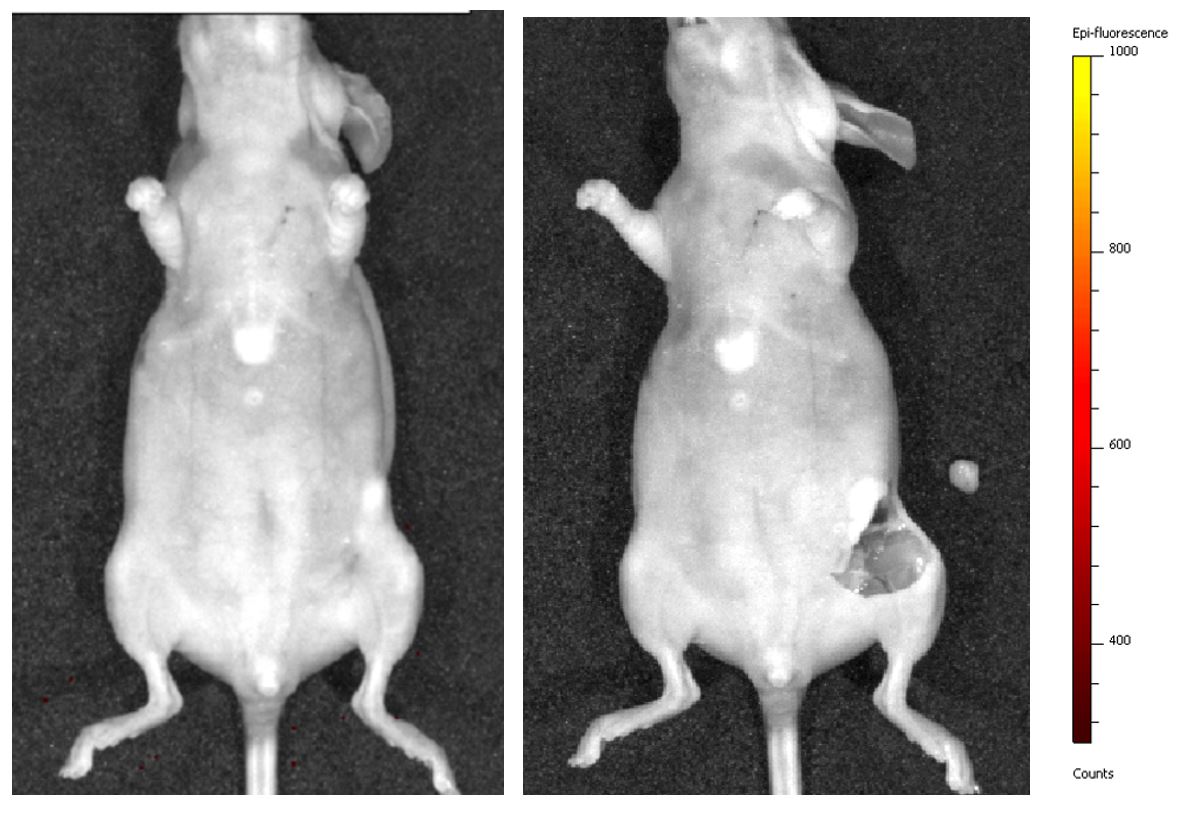

Supplement: Supplementary file 1 — Additional file 1: Orthotopic breast tumor model. During the period of tumor inoculation 8 mice developed abdominal skin lesions and had enlarged abdomens. After dissection of two of these mice, no clear indications were seen for these symptoms. The quantification of tumor uptake was unreliable in 2 mice because the tumor weight could not be determined accurately (below 0.01 gram). Two tumor measurements were left out of the analysis due to a substantial deviation of the average %IA/g: 6 %IA/g tumor uptake after injection of 10 μg and 30 %IA/g tumor uptake after injection of 30 μg. Both measurements were > 9 SD from the average in their group. One error could be explained by a substantially lower tumor weight and almost no HER2 expression (confirmed by immunohistochemistry); for both mice there was no difference in uptake of the tracer in other organs. Figure S1. Fluorescence images before and after resection of an orthotopic MCF7 tumor (A + B), 48 hours after administration of 30 μg [111In]In-DTPA-trastuzumab-IRDye800CW. The MCF7 tumor shows no fluorescence signal on the selected epi-fluorescence scale of 300-1000 counts. [file 13550_2019_564_MOESM1_ESM.jpg]
